# Controlling factors for shale gas enrichment and their implications for favorable exploration areas: Insights from the Wufeng–Longmaxi Formations, Southern Sichuan, China

**DOI:** 10.1371/journal.pone.0323277

**Published:** 2025-05-23

**Authors:** Zhenwei Zou, Jinghao Fu, Hu Li, Yongyang Liu, Chenglin Zhang, Ziqiang Xia, Cunhui Fan

**Affiliations:** 1 School of Geoscience and Technology, Southwest Petroleum University, Chengdu, Sichuan, China; 2 School of Economics, Sichuan University of Science and Engineering, Yibin, China; 3 Shale Gas Research Institute, PetroChina Southwest Oil & Gas Field Company, Chengdu, China; Hefei University of Technology School of Resources and Environmental Engineering, CHINA

## Abstract

The exploration and development of shale gas in the Wufeng-Longmaxi Formations (WF-LMX FM) of the Southern Sichuan Basin exhibit pronounced spatial heterogeneity with 3–5 fold gas content variations in strata meeting identical static thresholds (TOC > 2.5wt.%, porosity >5%). This exposes a fundamental disconnect in conventional models that dissociate geochemical potential from tectonic preservation dynamics in post-mature shale systems (Ro > 3.0%). This spatiotemporal decoupling is resolved through process-contingent integration of three critical determinants: thermal maturation trajectories, hydrocarbon generation-preservation windows, and multi-phase tectonic stress imprinting, with fracture connectivity enhancement observed at reduced brittleness thresholds (~35%) via pre-existing microfracture networks inherited from multi-phase deformation events. This study establishes a pioneering multivariate evaluation framework integrating well logs, 3D seismic interpretation, and experimental petrology to decode five governing parameters: Total Organic Carbon (TOC), thermal maturity (Ro), porosity, mineral brittleness, and a preservation potential index (SP) that resolves TOC-Ro-gas content decoupling by quantifying fault throw/erosion rate equilibrium calibrated with shale bed thickness anomalies. Systematic analysis reveals that optimal enrichment necessitates threshold recalibration beyond static paradigms-TOC > 4.0wt.% redefines hydrocarbon potential in high-maturity regimes, while the 3.0-4.0% Ro window balances organic porosity evolution against carbon deposition-induced occlusion. Reservoir viability is further constrained by >4.5% porosity for gas storage capacity and >40% brittle minerals for fracture sustainability. The SP index, incorporating erosional magnitude and fault connectivity, identifies two critical risk zones near the Changning anticline and northern Luzhou, where neotectonic fault reactivation disrupts overpressure maintenance. Spatial synthesis of these constraints delineates two strategic exploration targets: the NW-SE trending Jianwu-Weiyuan slope system exhibiting optimal thermal-structural synergy, and the Luzhou northern anticlinal cluster where fault sealing sustains preservation efficacy. This model resolves the “geochemical preservation paradox” through dynamic process coupling, establishing a transformative paradigm for deep shale gas exploration in thrust-fold terrains.

## 1 Introduction

In recent years, the exploration and development of shale gas in the Wufeng-Longmaxi Formations (WF-LMX FM) of the Sichuan Basin has achieved large-scale industrialization, with areas such as Weiyuan, Fuling, Luzhou, Changning, and Zhaotong emerging as critical zones for shale gas resource exploitation in China [1–5]. Globally, shale gas development has evolved into a diversified landscape characterized by area-specific geological and technological frameworks.

As the birthplace of the shale gas revolution, North America has dominated this sector, exemplified by the Marcellus, Haynesville, and Barnett shale plays in the United States. Technological breakthroughs in hydraulic fracturing and horizontal drilling propelled U.S. natural gas production to global leadership, with shale gas contributing over 80% of the nation’s total natural gas output in 2022. In Canada, the Montney and Duvernay shale formations have achieved commercial viability through multi-stage fracturing technologies, boasting proven reserves exceeding 1.2 trillion cubic meters (TCM). In South America, Argentina’s Vaca Muerta shale, with estimated resources surpassing 2.3 TCM, has become the Southern Hemisphere’s most promising shale gas basin, achieving a daily production milestone of 40 million cubic meters (Mm³/d) in 2023. European basins, such as Poland’s Baltic and the UK’s Bowland shales, have confirmed the presence of medium-to-high maturity shale gas reservoirs despite regulatory constraints on environmental grounds [6–10]. Australia’s Cooper Basin has economically exploited low-permeability shale gas through advanced “sweet spot” identification technologies.

Table 1 provides a comparative analysis of key geological parameters between the Longmaxi Formation (LMX FM) in southern Sichuan and globally representative shale gas plays [9–11]. The LMX FM exhibits a distinct geological profile marked by high thermal maturity (Ro), overpressure systems, and heterogeneous reservoir properties. These characteristics contrast sharply with the “high-silica, moderate-maturity” model typifying North American shales, necessitating tailored engineering strategies for efficient resource extraction.

**Table 1:** Comparison of key parameters between LMX FM and major global marine shale Formations (Barnett, Marcellus, Vaca Muerta).

Parameters	LMX FM	Marcellus Shale (USA)	Marcellus Shale (Canada)	Vaca Muerta Shale (Argentina)
Geological age	Silurian	Devonian	Triassic	Jurassic-Cretaceous
TOC (wt.%)	2.99-3.78	3-12	1-3	4-8
Ro (%)	2.38-3.37	1.5-3.0	1.0-1.8	0.9-1.5
Reservoir thickness (m)	26-70	15-60	300-600	300-500
Pressure coefficient	1.2-1.6	1.1-1.3	1.1-1.3	1.2-1.5
Burial depth (m)	2000-4000	1200-2600	2000-3500	2500-4000
Clay mineral content (%)	15-40	10-30	20-40	20-35

The W201 well, China’s first shale gas well, was successfully fractured and achieved industrial gas flow in 2010, marking a historic milestone in the country’s shale gas exploration. In August 2011, China’s first horizontal shale gas well, W201-H1, was brought into production, with a daily output surpassing 10,000m^3^. Currently, the proven reserves of LMX shale gas in the southern Sichuan Basin, at depths of less than 3500m, are estimated to be 2.96 TCM [12]. In the Changning shale gas demonstration area, daily production has exceeded 20Mm³/d, making it the highest in China in terms of daily shale gas production, highlighting the area’s excellent preservation conditions and promising exploration prospects [13].

Prior research has conducted a systematic analysis of the shale gas in the WF-LMX FM of the southern Sichuan area, including aspects such as sedimentary environment, reservoir characteristics, gas content, organic matter abundance, and type, as well as the key factors controlling enrichment [14–18]. Based on these analyses, multiple key theories have been formulated. Among them, the “dual enrichment” rule emphasizes the importance of high-quality mudstone and favorable preservation conditions as key factors for efficient shale gas enrichment [19]. The “three controls” theory for high-yield enrichment is refined into three main factors: sedimentary and diagenetic process control, reservoir control, and structural and preservation condition control, all of which are core to shale gas enrichment and high yield [20]. The “source-seal control” theory clarifies that effective source rocks and cap layers are essential prerequisites for the formation of shale gas reservoirs. A wealth of theories and models has provided scientific guidance for the key shale gas control enrichment conditions, including hydrocarbon sources, reservoir formations, and preservation, effectively advancing exploration and development activities in the southern Sichuan area [21,22]. This study focuses on the WF-LMX FM in the southern Sichuan area, utilizing available data from seismic surveys, drilling, core sampling, and well logging to analyze factors such as hydrocarbon source rock conditions, reservoir characteristics, and integrated preservation conditions. The paper also examines areal differences in LMX shale gas reservoirs across southern Sichuan, further deepening the understanding of high-yield shale gas characteristics in the area. It provides technical support for selecting shale gas layers in the LMX FM, as well as establishing criteria for layer selection and parameter values, to advance current exploration of the LMX FM toward the development of new stratigraphic layers.

## 2 Geological setting

The Sichuan Basin is a large, multi-phase composite basin situated on the Upper Yangtze Craton, and has undergone complex geological evolution, resulting in its distinctive rhombic shape. This structure is primarily controlled by the intersection of deep, large-scale faults trending northeast-southwest and northwest-southeast within the Upper Yangtze Craton. Following the late Ordovician Hirnantian glaciation, the Sichuan Basin underwent the formations of the Sichuan and Qiannan paleo-uplifts at its basin margins [23–25]. The basin evolution was subsequently controlled by the Chuanzhong Caledonian Paleo-Uplift during the Yanshanian tectonic movement (Jurassic-Cretaceous), which established a semi-restricted marine environment characterized by low-energy hydrodynamic conditions, sediment undercompensation, and pervasive shelf hypoxia. This structural framework, coupled with two global marine transgressions (late Ordovician to early Silurian) driven by eustatic and tectonic forcings, promoted the deposition of organic-rich shales within the LMX FM [26,27].

**Fig 1:**
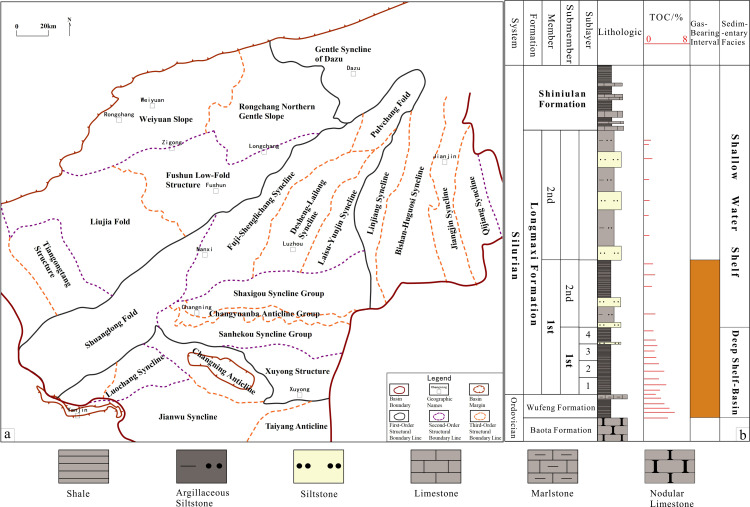
Geological setting of the southern Sichuan Basin. a. Structural configuration; b. Stratigraphic distribution of the WF-LMX FM.

The southern Sichuan area, influenced by multiple tectonic events, displays an overprinted and composite structural framework, with NE, WE, and nearly SN-oriented structures [28,29]. It is characterized by broom-like low-angled anticlines, with structural forms becoming progressively broader and less folded from north to south (Fig 1a). Based on the basement structure, fault and fold morphology, structural style, and tectonic evolution phases, the current base of the Wufeng Formation (WF FM) in southern Sichuan can be classified into five first-order structural units, eleven second-order structural units, and twenty-five third-order structural units [30]. In these structural units, the burial depth of third-order units such as the Weiyuan slope, Changning anticline, Jianwu syncline, and Taiyang anticline is less than 3500m, while the burial depths of other units generally exceed 3500m, classifying them as deep to ultra-deep formations.

The study area lies in the southwestern part of the Sichuan Basin and its surrounding area, spanning the middle slope low-fold zone of southwestern Sichuan and the southern Sichuan paleo-depression mid-uplift low-angle dome zone. It is influenced by the combined control of the Jiangnan Xuefeng Uplift Belt, the Qiannan Uplift Belt, the Kongdian-Dianxi ancient continental block, and the Sichuan paleo-uplift [31]. The southern Sichuan area and its basin margin are intersected by the Changshou-Zunyi Fault Zone, the Gulin-Yanjin Fault Zone, and the Huaying Mountain-Qingshan Ridge Fault Zone, forming a geomorphic feature with a right-angled triangular boundary in the south.

The sedimentary environment of the LMX FM in southern Sichuan formed in a deep marine shelf environment during the late Ordovician to early Silurian. During this period, the basin underwent rapid marine transgression, with the depositional setting shifting toward a low-energy, deep-water anoxic environment [32,33]. These stable, enclosed marine conditions effectively inhibited the oxidation and decomposition of organic matter, allowing for the accumulation of large quantities of organic matter in the sediments, creating an ideal “primary hydrocarbon source”.

The LMX FM in southern Sichuan is divided into the Long-1 and Long-2 Members from bottom to top. This study focuses on the 1st Submember of Long-1 Member, which includes the first, second, third, and fourth Submembers, mainly consisting of clay shale, siliceous shale, sandy mudstone, and marl limestone (Fig 1b). The siliceous shale at the base of the LMX FM is particularly notable, with high organic carbon content, which is a “gift” of this unique sedimentary environment [34].

## 3 Samples and methods

### 3.1 Samples

This investigation conducted systematic core analysis on 9 key wells in the Sichuan Basin, with continuous coring lengths as follows: N201 (43.25 m), N213 (60.5 m), N216 (50 m), N218 (43 m), Y205 (36 m), Y202 (45 m), Y206 (35 m), Y207 (79 m), and Y208 (33 m). All samples were derived exclusively from core specimens, with no outcrop samples included in this study. The sampling strategy focused on the middle-lower members of the Upper Ordovician WF FM through Lower Silurian LMX FM in 7 wells (L203H79-4, L205, Y101H10-3, etc.), including 24 samples for TOC analysis from representative wells (N201, N213, Y205, Y207, Y208), 25 samples for solid bitumen reflectance across structural domains (anticlinal cores in N216 and Y202; synclinal flanks in Y206), and 20 shale samples for SEM-EDS characterization encompassing dominant lithofacies (siliceous shale: 13 samples, 65%; calcareous shale: 7 samples, 35%).

### 3.2 Methods

#### 3.2.1 Total organic carbon.

The TOC content of 24 shale samples was analyzed using a CS-230 carbon-sulfur analyzer to evaluate organic matter abundance and hydrocarbon generation potential. In strict accordance with the national standard GB/T 19145–2003 (Determination of TOC in Sedimentary Rocks), this method integrates high-temperature combustion (1200 °C) under oxygen-rich conditions with infrared spectroscopy for precise carbon quantification. Samples were pulverized to ≤200 mesh (≤75 μm) using a tungsten carbide ball mill to ensure homogeneity and complete combustion efficiency, adhering to standardized preparation protocols. Approximately 50 mg of homogenized powder was combusted in a horizontal tube furnace with continuous oxygen flow (200 mL/min), converting organic carbon to carbon dioxide (CO_2_). Evolved CO_2_ was transported via helium carrier gas to a dual-wavelength infrared detector (4.26 μm and 2.7 μm absorption bands) for real-time quantification. Quality control measures mandated by GB/T 19145–2003 were implemented throughout, including triplicate analyses of certified calcium carbonate standards (0.1-20 wt% carbon) to establish linear calibration curves (R^2^ > 0.999) and ensure ±0.1 wt% measurement precision.

The integrated data analysis software processed spectral signals and generated TOC reports, providing quantitative metrics for organic carbon enrichment levels. Replicate analyses (N = 5) demonstrated high reproducibility (relative standard deviation <3%), while thermal gravimetric cross-validation confirmed >95% recovery efficiency. These rigorously validated TOC data not only characterize organic carbon distribution but also establish a robust experimental foundation for assessing source rock hydrocarbon potential and its correlation with reservoir properties [35,36].

#### 3.2.2 Solid bitumen reflectance determination and empirical conversion to equivalent vitrinite reflectance in pre-devonian strata.

Thermal maturity assessment of 25 samples in this study was performed through reflectance measurements of organic macerals using a Leica DM4500P microscope equipped with a 546 nm monochromatic light source and automated photometer. Samples were initially cut into 3–5 mm thick slabs using a diamond wafering blade, followed by epoxy resin impregnation under vacuum (25 kPa) at 60°C for 48 hours to ensure complete pore-filling and structural stabilization. For Pre-Devonian samples devoid of true vitrinite (absent before vascular plant evolution), reflectance values were obtained from solid bitumen particles and converted to equivalent vitrinite reflectance (EqVRo) using the empirical correlation [37,38]:


$$EqVRo=0.618\times BRo+0.40(R^{2}=0.91,n=120),$$

where BRo denotes solid bitumen reflectance. Post-Devonian samples were analyzed for true vitrinite reflectance following ISO 7404–5 protocols. The impregnated slabs were ground sequentially with 600-, 1200-, and 4000-grit silicon carbide paper, then polished with 0.05 μm alumina suspension to achieve optical-grade surfaces. Polished blocks (3–5 μm thickness) were prepared under oil immersion (n = 1.518 at 23°C). A minimum of 50 homogeneous particles per sample were analyzed at 50 × magnification, excluding fissures and mineral inclusions.

Instrument calibration utilized synthetic spinel (0.42% Ro) and yttrium-aluminum-garnet (0.90% Ro) standards, achieving ±0.05% reproducibility. Outliers (>2σ) were removed via Grubbs’ test (95% confidence). For Pre-Devonian strata, the derived EqVRo values (0.8–3.2%) represent thermal maturity proxies, acknowledging a systematic overestimation of 0.2–0.5% at BRo > 2.0% due to bitumen graphitization. Maturity stages were classified as immature (<0.5%), oil window (0.5–1.3%), wet gas (1.3–2.0%), and dry gas (>2.0%). Cross-validation with Rock-Eval T_max_ data (r = 0.89, p < 0.001) confirmed methodological robustness. These calibrated maturity indices provide critical constraints for modeling hydrocarbon generation kinetics and reservoir quality evolution in shale systems.

#### 3.2.3 Scanning electron microscope observation.

In this research, a Hitachi field emission SEM was employed to analyze the microstructure of 20 shale samples, observing and qualitatively analyzing the organic matter and pore structure [39]. The device features a cold field emission system integrated with an Oxford X-MaxN 150 mm² silicon drift detector (EDS), allowing for high electron beam brightness and stability at lower voltages (1.2 nm resolution at 15 kV), thus providing imaging with ultra-high resolution calibrated daily using NIST SRM 2090 certified reference material. The SEM magnification range was set from 350x to 16,000x with a pixel resolution of 0.8nm at 16,000 × magnification, allowing for flexible observation at both macro and micro levels, capturing surface details of the samples through 3-stage Au-Pd sputter coating (5nm thickness) and producing clear images of the microstructure with optimized dwell time (20 μs/pixel) and 10-frame averaging for noise reduction.

In the experiment, secondary electron probes recorded the surface structure of the samples, and energy-dispersive X-ray spectroscopy (EDS) was used for semi-quantitative analysis of the elemental composition, precisely identifying the chemical constituents and distribution of organic matter and inorganic matrix in the samples. These high-resolution imaging and elemental analysis data reveal the distribution patterns, morphology, and connectivity of organic matter within the pores, providing a deeper understanding of its relationship with the inorganic matrix, and offering reliable data support for research into shale reservoir characteristics and hydrocarbon generation potential.

## 4 Identification of the key controlling factors for shale gas accumulation in the South Sichuan area

### 4.1 Integrated assessment of hydrocarbon source rock conditions

Favorable source rock conditions form the foundation for large-scale and continuous oil and gas accumulation. As an unconventional energy source, shale gas, which is self-generated and self-stored, has its accumulation primarily controlled by its material foundation, reservoir structure, and subsequent preservation conditions. The material foundation of shale not only plays a crucial role in the reservoir’s hydrocarbon generation potential but also impacts the pore space of the reservoir, thereby influencing its gas content. The study of shale’s material foundation primarily revolves around the analysis of shale thickness, TOC content, and Ro Efficiency as key evaluation parameters. This study will assess the organic matter abundance in the South Sichuan area by focusing on two critical indicators: TOC and Ro.

#### 4.1.1 Total organic carbon.

TOC content is a critical factor in the enrichment and accumulation of shale gas, serving as a fundamental parameter for evaluating reservoir quality and gas storage capacity. In the area, the spatial heterogeneity of TOC deposition controlled by paleo-productivity, redox conditions, and sediment dilution rates directly governs gas generation potential and storage efficiency [40,41]. For instance, in deep-water shelf environments (e.g., the WF-LMX FM), sustained anoxia promoted organic matter preservation, resulting in high TOC layers (3–5wt.%) that enhance methane adsorption capacity by 20–40% compared to low-TOC intervals (<2wt.%) [42]. These organic-rich facies exhibits dual gas storage mechanisms: (1) adsorbed gas dominantly hosted in organic matter nanopores (pore size <10 nm), and (2) free gas accumulated in fractures and interparticle pores [43,44].

The coupling between TOC distribution and Ro further modulates gas storage patterns. In high-maturity areas (Ro > 2.5%), excessive organic matter cracking reduces micropore volume by 15–30%, diminishing adsorption capacity despite elevated free gas fractions. Conversely, moderate maturity (Ro = 1.3–2.0%) optimizes the coexistence of adsorbed and free gas, as observed in the Marcellus Shale’s gas storage model. Such areal variations in organic carbon deposition necessitate basin-specific evaluation frameworks to predict gas-in-place and prioritize drilling targets.

**Fig 2:**
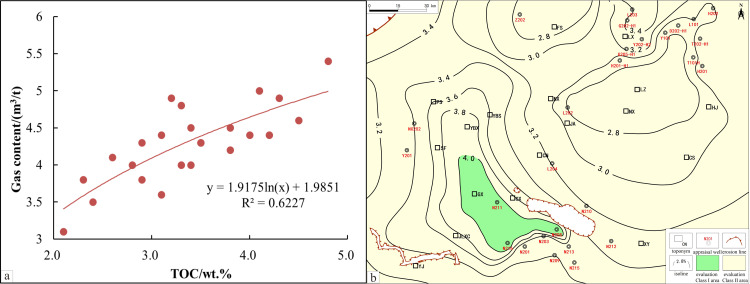
TOC isoline evaluation map and its correlation with gas content. a. TOC isoline evaluation map of the study area. b. correlation between TOC evaluation results and gas content.

A comparison of field data from the LMX FM shale in the South Sichuan area shows a distinct positive correlation between TOC and total gas content in the evaluation wells: higher TOC values correlate with better gas content (Fig 2a) According to national standards, shale gas content above 2.0 m^3^/t is regarded as commercially exploitable. When TOC is below 1.0wt.%, shale gas content is typically less than 2.0 m^3^/t. Thus, in the South Sichuan area, adjusting the lower limit of TOC to 1.0wt.% better suits the local geological conditions and practical requirements.

Based on the development risks of various TOC intervals, this study recommends categorizing the 1.0wt.%-2.0wt.% range as a high-risk development area, the 2.0wt.%-4.0wt.% range as a more favorable exploration and development area, and areas with TOC over 4.0wt.% as preferred development zones. Using the aforementioned TOC parameter thresholds (Fig 2b), areas with high TOC levels are mainly concentrated within the Luochang and Jianwu Synclines, with some areas extending upward into the Sanhekou syncline group structure.

#### 4.1.2 Thermal maturity.

Ro is a crucial parameter for shale gas accumulation and enrichment. It influences the phase state, hydrocarbon generation, and the shape and depth of the storage space in the formations, while also playing a pivotal role in the formation and development of micro- and nanoscale pores. Therefore, Ro is a key resource potential parameter in shale gas site evaluation [45,46].

According to the oil generation theory, for Type I and II kerogens, when Ro exceeds 0.5%, the kerogen enters the oil generation phase. Once Ro reaches 1.3%, the kerogen starts to degrade and generate gas, with liquid hydrocarbons potentially undergoing secondary cracking into gas. These transition points are critical factors to consider when assessing the shale gas resource potential. Therefore, for the marine shale gas site selection evaluation in the South Sichuan area, the lower limit of Ro is defined as 1.3% [47].

**Fig 3:**
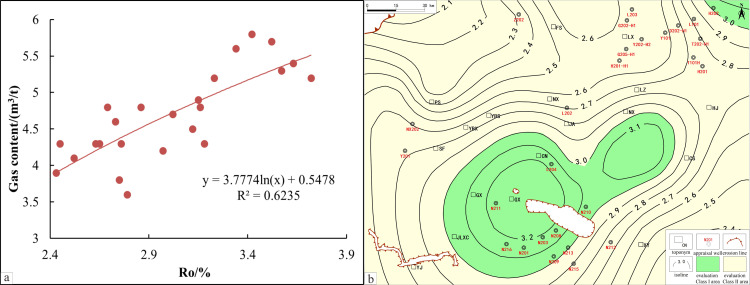
Ro isoline evaluation map and its correlation with gas content. a. Ro isoline evaluation map of the study area. b. correlation between Ro evaluation results and gas content.

A comparison of actual production data from typical wells in the South Sichuan area with Ro reveals a significant positive correlation between the two (Fig 3a). Based on this, and supported by research from both domestic and international scholars, Ro = 1.3% is established as the lower limit for site evaluation. Areas with Ro values between 3.0 and 3.5 are considered favorable for development, while areas with Ro values between 2.0 and 3.0 are regarded as core favorable zones. However, excessively high Ro values may lead to issues in the shale gas accumulation process, such as gas depletion, organic matter carbonization, and pore closure, etc As a result, Ro = 4.0 is defined as the upper maturity limit for the calcareous-argillaceous shales of the Sichuan Basin, where high carbonate content (40–54%) accelerates diagenetic pore occlusion at elevated Ro. This threshold may not universally apply to other basins; for instance, silica-rich shales (e.g., Duvernay Formations, Canada) sustain gas production at Ro > 4.5% due to enhanced brittleness delaying pore collapse while clay-dominated systems (e.g., Bowland Shale, UK) require lower thresholds (Ro < 3.5) to mitigate ductility risks [48–51].

A Ro evaluation map for the South Sichuan area was constructed based on the above-established criteria (Fig 3b). The results indicate that the selected favorable areas span across the Luochang syncline, Changning anticline, and Sanhekou syncline group, and extend northeastward into the Linjiang syncline structural zone.

### 4.2 Reservoir conditions analysis

Reservoir performance plays a crucial role in shale gas enrichment and accumulation, as its quality directly influences the storage characteristics and recoverability of gas in the reservoir. In shale gas, self-generated and self-stored unconventional energy, porosity, and brittle mineral content are the core parameters for evaluating reservoir performance [18,52–55]. They jointly affect the fracturing potential and gas flow efficiency of the reservoir.

Porosity is a fundamental indicator of a reservoir’s gas storage capacity. The pore system in shale reservoirs is complex and diverse, comprising organic matter pores, intergranular gaps, and natural fracture pores. The combined effects of these different pore types shape the overall gas storage potential of the reservoir. High porosity indicates the reservoir can store more free and adsorbed gas, sustaining long-term effective accumulation. In contrast, the content of brittle minerals is a key indicator of a reservoir’s fracturing potential. Reservoirs rich in brittle minerals, such as quartz and feldspar, are more prone to forming complex fracture networks under external forces [56,57]. This feature significantly improves the reservoir’s permeability and conductivity, allowing for smoother gas flow and enhanced recovery efficiency. Conversely, if the brittle mineral content is insufficient, the reservoir will fail to generate effective fractures during the fracturing process, restricting gas release and flow, which ultimately affects production efficiency.

#### 4.2.1 Porosity.

**Fig 4:**
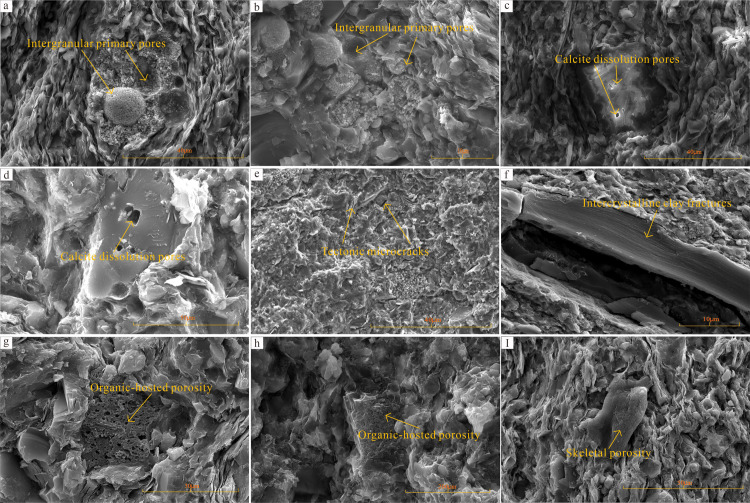
Reservoir space types of the WF-LMX FM. a. L203H79-4 well, LMX FM, 3786.15 m: Framboidal pyrite is present, indicative of the development of residual primary pore spaces. b. L203H79-4 well, LMX FM, 3836.11 m: Organic matter, dolomite crystals, and mica fragments are observed, demonstrating intergranular porosity between organic matter and minerals. c. L203H79-4 well, LMX FM, 3856.17 m: Partially dissolved calcite pores are filled by microparticulate calcium feldspar and kaolinite, representing mineral dissolution porosity. d. L205 well, WF FM, 3962.61 m: Extensive interlaminar fractures in clay minerals are developed, characteristic of the clay interlayer-type reservoir. e. Y101H10-3 well, LMX FM, 3752.84 m: Organic matter displays a film-like distribution with the development of primary biogenic pore spaces. f. L211 well, LMX FM, 4790.39 m: Significant organic matter porosity is evident, typical of the organic pore-type reservoir. h. L206 well, LMX FM, 3969.73 m: Dissolution porosity is developed, evidenced by microparticulate calcite filling. i. L206 well, LMX FM, 4014.79 m: A widespread distribution of large framboidal pyrite is observed, accompanied by the formations of interlayer microfractures.

Porosity is a crucial aspect of reservoir evaluation and an essential parameter in the calculation of oil and gas reserves. Pores serve as the main location for the free storage of shale gas, and porosity directly governs the proportion of free gas within the shale. Shale gas can only reach industrial production when porosity meets certain conditions, making porosity a critical parameter for evaluating the storage capacity of reservoirs in shale gas selection [2,4]. Based on core observation, microscopic identification, and whole-rock diffraction analysis, the genetic classification of marine shale reservoirs was conducted by considering factors such as diagenesis, deep-burial processes, and quasi-metamorphism. These reservoirs were categorized into four main types: matrix porosity-type, clay interlayer-type, organic pore-type, and biogenic pore-type (Fig 4) [58–60].

The matrix porosity-type reservoir comprises storage spaces typical of conventional oil and gas reservoirs, including intragranular pores, intergranular pores, and microfractures commonly observed in clastic and carbonate rocks. Based on pore geometry, these are classified as residual primary pores (Fig 4a, b), stable mineral dissolution pores (Fig 4c, d), and tectonic microfractures (Fig 4e). The clay interlayer-type reservoir develops during diagenesis when clay minerals in shale undergo dehydration and transformation. This process alters their structural and chemical properties, releasing significant amounts of interlayer and structural water, and thereby generating abundant clay mineral interlayer fractures (Fig 4f). The organic pore-type reservoir (Fig 4g, h) predominantly occurs in strata with high TOC content. In the high-overmature stage, extensive thermal degradation and pyrolysis of organic matter lead to substantial hydrocarbon expulsion and the formation of micropores ranging from the micrometer to the nanometer scale, with pore diameters concentrated between 2 and 50 nm. The biogenic pore-type reservoir (Fig 4i) originates from inherent pores within organic hydrocarbon-generating precursors, such as body cavity pores in lower organisms (e.g., foraminifera) and tissue pores in higher plants (e.g., vascular bundles), all of which are classified as primary pores.

It is widely accepted by scholars and industry practitioners, both in China and abroad, that the lower limit for porosity in shale reservoirs should be 2.0%. Below this “red line”, the commercial exploitation potential of shale gas is significantly diminished [61–63]. In deep reservoirs (>3,500 m), permeability compensation mechanisms overcome this constraint through synergistic geological and engineered interactions. Structural deformations create brittle-ductile transition zones where interconnected fracture networks enhance permeability by 10–100 × compared to undeformed strata at equivalent porosity levels, effectively bypassing matrix limitations. During production, pressure depletion triggers matrix shrinkage (0.1-0.3% strain) and desorption-enhanced pore connectivity, generating self-reinforcing flow pathways that amplify permeability in nanopore systems under confining pressures >50 MPa. Advanced hydraulic fracturing optimized for depth conditions (e.g., cluster spacing <15 m at 4,000 m) creates complex fracture networks with permeability exceeding 700 mD-three orders of magnitude above matrix values-while supercritical water treatments (380°C, 23 MPa) demonstrate transformative potential by enhancing permeability-porosity ratios through organic-inorganic reactions, achieving 2,198% improvement in ultra-low porosity shales (1.2-1.8%). These mechanisms operate dominantly in overpressured systems where gas mobility (Knudsen numbers >0.1) couples with stress-sensitive fracture conductivity to govern flow dynamics. Crucially, successful compensation requires reservoirs to meet specific geological criteria: > 30% brittle minerals to sustain fracture networks, TOC > 2wt.% for sufficient gas generation, and <30% clay content to prevent pore-throat occlusion. The commercial viability of sub-2% porosity shales thus hinges on the precise integration of reservoir-specific stress regimes, mineralogical constraints, and engineered stimulation strategies that exploit these depth-enhanced permeability synergies.

**Fig 5:**
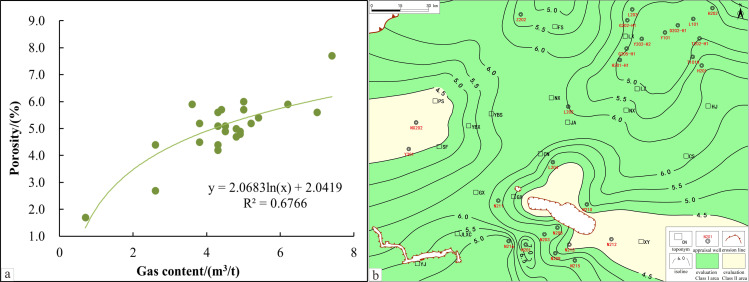
Porosity isoline evaluation and its correlation with gas content. **A.** Porosity isoline evaluation. **B.** Correlation between porosity and gas content.

Integration of actual production data and field porosity data reveals a significant positive correlation between porosity and gas content (Fig 5a). Based on this observation, and using the porosity classification standards proposed by researchers, areas with porosity below 2.0% are categorized as low-grade, unfavorable, and low-scoring areas, while those with porosity between 2.0% and 4.5% are primarily classified as medium-grade, relatively favorable, and medium-scoring zones. As a result, 2.0% is set as the lower limit for porosity, to more accurately identify and evaluate potential commercial extraction areas, without generally setting an upper limit for porosity. Based on this standard, the porosity of the LMX FM reservoirs in the southern Sichuan area generally exceeds 4.5%, demonstrating the potential for the development of extensive and continuous reservoirs (Fig 5b).

#### 4.2.2 Brittle mineral.

Brittle mineral content is crucial in determining the matrix porosity, microfracture development, gas-bearing characteristics, and reservoir stimulation methods of shale, and it also impacts the formations of natural fractures. The high content of quartz, feldspar, and carbonates, along with the low content of clay minerals in shale, increases its brittleness, making it more prone to the formation of natural and induced fractures under external forces, thus facilitating reservoir fracturing and productivity enhancement [64,65]. The brittleness of shale reservoirs is directly influenced by the content of brittle minerals, which increases the brittleness index, and Young’s modulus, and decreases Poisson’s ratio, thus facilitating easier fracturing [55,66]. Quartz and carbonate minerals make formations more susceptible to brittle fractures, aiding in fracturing operations, while clay minerals, which do not easily react with fracturing fluids, hinder the fracturing process. Thus, the brittle mineral content is a critical parameter in evaluating shale gas exploration areas.

In North American shale gas exploration areas, quartz content typically ranges from 28% to 52%, carbonate mineral content from 4% to 16%, with total brittle mineral content (quartz + carbonate + feldspar) reaching up to 70%. The minimum threshold for commercial development of brittle minerals is generally set at 40%, a standard regarded by Schlumberger and Halliburton as a core indicator of fracturing effectiveness [67]. However, global variations in shale mineral composition reveal the areal limitations of this threshold.

For example, in the LMX FM of the Sichuan Basin, quartz content (35–48%) is comparable to that of the Marcellus Shale in North America (28–52%), but carbonate mineral content (8–15%) is significantly lower than in the Haynesville Shale (16–25%) [68]. Although total brittle mineral content reaches 43–63%, actual fracturing efficiency relies more on the quartz-to-clay mineral ratio (>1.5:1) rather than total brittleness, indicating that rigid application of the 40% threshold may underestimate the regulatory role of mineral assemblages [69].

In contrast, the Vaca Muerta Shale in Argentina exhibits substandard quartz content (20–35%) but elevated carbonate content (15–30%). Enhanced shear-slip effects in natural fractures enable reservoirs with 35–65% brittle minerals to achieve economic productivity of 2 × 10⁴ m³/d, further challenging the universality of rigid thresholds. Notably, the Baltic Basin in Poland, with brittle mineral content mostly below 35%, overcomes matrix brittleness limitations through optimized artificial fracture network complexity (fractal dimension >1.8) [69], highlighting the potential of integrated geoengineering technologies to expand threshold applicability.

The geological specificity of the North American threshold lies in its high quartz proportion (60–80% within brittle minerals), whereas the LMX FM and Vaca Muerta Shale exhibit quartz proportions of 55–70% and 40–55%, respectively [70,71]. Mineralogical heterogeneity results in divergent fracture propagation patterns under equivalent brittle mineral content: high-quartz systems in North America favor planar fracture development, while areas with carbonate content >20% require proppant concentration increases (from 2 kg/m² to 5 kg/m²) to maintain conductivity.

Consequently, brittle mineral thresholds must integrate areal mineral assemblages and thermal evolution history. For instance, quartz cementation in overmature Sichuan Basin shales (Ro > 2.5%) strengthens natural fracture networks, allowing operational thresholds to be reduced to 35%, whereas North American mid-maturity shales (Ro = 1.3–2.0%) strictly rely on brittle mineral support in artificial fracture systems.

**Fig 6:**
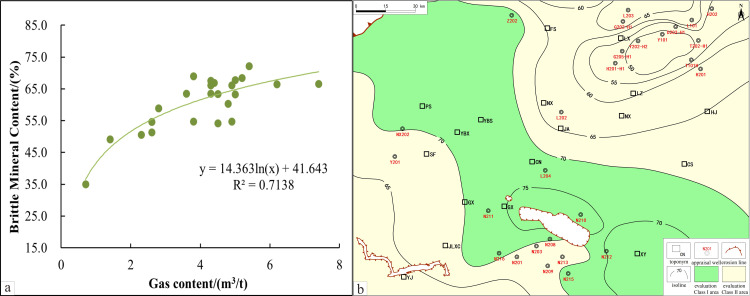
Brittle Mineral content isoline evaluation and its correlation with gas content. **a.** Brittle mineral content isoline evaluation. **b.** Correlation between brittle mineral content and gas content.

An analysis of production data in the southern Sichuan area reveals a strong positive correlation between brittle mineral content and the total gas content in the reservoir (Fig 6a). This positive relationship reinforces the crucial role of brittle minerals in shale gas accumulation and capacity improvement. Higher brittle mineral content not only improves the fracture formation capability of the reservoir during hydraulic fracturing but also promotes the desorption and flow of adsorbed gas in the shale matrix, thereby enhancing the gas production performance of individual wells. In contrast, shales with higher clay mineral content, due to their plastic properties, make it more challenging to form an effective fracture network during fracturing, restricting the reservoir’s permeability and gas recovery rate [72].

According to North American experience, brittle mineral content in shale reservoirs should exceed 40% to ensure effective fracturing and optimal production. Clay mineral content should stay below 30% to reduce the risks of plastic deformations and pore shrinkage, maintaining reservoir stability under pressure. Therefore, the brittle mineral content lower limit is set at 40% to balance fracturing efficiency with stability [73].

To visually depict the spatial variations in brittle mineral content within the WF-LMX shale of the southern Sichuan area, a zoning map was constructed (Fig 6b). The map illustrates that preferred areas with higher brittle mineral content are distributed from northwest to southeast, extending from the Weiyuan slope in the north to the Jiangu anticline and Taiyang backfold areas near the basin edge in the south.

### 4.3 Integrated analysis of preservation conditions

In the evaluation of marine shale gas reservoirs, key integrated evaluation factors for preservation conditions include effective shale continuous thickness burial depth, structural style, surface erosion degree, and pressure coefficient. Therefore, in the comprehensive evaluation of shale gas preservation conditions in the southern Sichuan Basin, reliance on a single factor to assess the overall preservation conditions of the study area should be avoided. On the contrary, reasonable evaluation standards should be set for each factor, and shale gas preservation conditions in the study area should be optimized through a comprehensive preservation condition evaluation grading standard.

In the southern Sichuan area, the structural styles and types of combinations display diverse and complex features, with significant development of structures associated with ductile shear deformations. These structures are primarily formed by the action of reverse faults, including fold deformations and associated structural types triggered by reverse faults, such as back thrust, thrust anticlines, imbricate fans, fault triangle zone, duplex structures, etc [74,75]. There is a marked lack of coordination in the expression of shear deformations between deep and shallow layers: deep shear layers show diverse deformations types and complex structural forms, while the bottom deformations layers typically have not undergone large-scale deformations and only retain remnants of early faults in local areas. The middle and shallow deformation layers exhibit more unstable deformation characteristics. This inconsistency in deformations is particularly evident in the horseshoe-shaped area in the northern Huaying Mountains tectonic belt, central and northern Yongchuan tectonic belt, and eastern Chishui tectonic belt. In contrast, the deep, middle, and shallow shear layers in the southern part of the study area show better coordination, with the extent of the deformation closely related to the thickness distribution of the shear layers.

The shear action caused by tectonic movements not only affects the development of faults but also directly influences the fault displacement, thereby impacting the preservation conditions of gas reservoirs. Typically, the greater the fault displacement, the larger the contact area between the shale layer and adjacent strata, the wider the fault zone, and the reduced clay content, which in turn weakens the sealing capacity of the fault. If the fault intersects permeable strata, it could result in lateral escape of shale gas. It is particularly noteworthy that when the fault displacement exceeds the thickness of the areal cap rock, the vertical extension of the fault is enhanced, and its sealing capacity is further reduced, thus increasing the risk of shale gas leakage.

Small-scale faults and fractures within the shale layer serve a distinct dual function in the storage and migration of shale gas. On the one hand, they can act as additional storage space, effectively promoting the desorption of adsorbed gas and providing local migration pathways, thus improving gas accumulation efficiency and enhancing the gas content of the shale layer. However, when these faults extend vertically and penetrate the upper and lower boundaries of the shale layer, they may become potential pathways for shale gas leakage. Particularly those faults that extend upward, cutting through the cap layer or even connecting to the surface, cause local pressure to drop sharply around these faults. As a result, the displacement pressure of the shale layer becomes significantly higher than that of the fault rock, leading to the loss of substantial shale gas through the fault pathways.

Under hydraulic fracturing conditions, the injection-induced pore pressure elevation can critically reduce the effective normal stress on pre-existing faults, triggering shear reactivation. This mechanical destabilization not only enhances fault permeability through dilation but also risks creating unintended conduits for gas migration, potentially connecting isolated fracture networks to larger fault systems [76–78]. Engineering mitigation requires maintaining injection pressures below the critical threshold for fault slip while ensuring sufficient fracture network complexity, necessitating advanced characterization of fault geometries and in-situ stress anisotropy to balance stimulation efficiency with subsurface integrity.

Based on the fault penetration depth and geological strata, the faults in the southern Sichuan area can be divided into four categories: the first category consists of faults within the WF-LMX FM, which primarily control the lateral migration of shale gas within the target layer [79]. The second category consists of faults extending into the Ordovician, which cut through the shale layers downward and help form deep composite reservoir spaces. The third category consists of faults extending upward into the Permian, and the presence of these faults may cause shale gas to escape through the overlying strata. The last category consists of faults that penetrate the surface, and their direct connection to the surface makes it easier for shale gas to escape, representing a key threat to the sealing capacity of gas reservoirs.

Additionally, changes in paleogeography and paleoclimate conditions further exacerbate the structural and stratigraphic complexity in the southern Sichuan area. The Caledonian orogeny at the end of the Late Silurian led to significant uplift and erosion in the area, especially in the Leshan-Longnüsi ancient uplift area, where most of the Upper Silurian strata were intensely eroded, leaving only the black shale at the base of the LMX FM. This erosion process did not cease with the Caledonian orogeny but intensified during the subsequent Yanshan-Himalayan period. Especially in the southern edge of the Sichuan Basin, due to continuous tectonic uplift, the erosion process intensified, eventually leading to the outcrop of the LMX FM black shale in the Longning-Xian County anticline belt [80].

Tectonic activities from the Indosinian to the Yanshan-Himalayan period further altered the burial pattern of the LMX FM. In the Luzhou ancient uplift and other elevated areas, intensified tectonic uplift led to an expansion of the erosion range, resulting in the partial exposure of the LMX FM at the margin. In contrast, the depressed zones between the ancient uplifts, due to weaker uplift, maintained greater burial depths. Well data and geological surveys indicate significant areal variations in the burial depth of the LMX FM in southern Sichuan, with an overall range from 0 to 5259 meters: burial depths near the southern part of Changning and the Long anticline near Gongxian are generally less than 2000 meters, while the depths in areas from Changning to Yibin, and from Chishui to Gulin, mostly exceed 4000 meters. This difference in burial depth directly affects the accumulation and preservation of shale gas in the LMX FM, further increasing the complexity of areal exploration.

In evaluating the shale gas preservation conditions of the LMX FM in southern Sichuan, this study comprehensively considers three key dimensions: areal tectonic evolution, effective traps, and favorable conditions for gas accumulation. Through multi-factor analysis, this study carried out a zonal evaluation of shale gas preservation conditions and developed a multi-tiered evaluation index system to assess the preservation conditions of shale gas in the LMX FM in Southern Sichuan (Table 2).

**Table 2:** Graded evaluation table of the comprehensive preservation conditions of shale in the WF-LMX FM of the Southern Sichuan area [81–83].

Evaluation parameters	Evaluation criteria			
	Favorable preservation area	Average preservation area		Poor preservation area
Structural deformations	dip angle	<10°	10 ~ 20°	>20°
Effective continuous shale thickness (m)	≥50	30 ~ 50		<30
Erosion thickness (m)	<2500	2500 ~ 3500		>3500
Pressure coefficient	≥1.2	0.8 ~ 1.2		<0.8
Distance to major fault (km)	>1.9	1.3 ~ 1.9		<1.3
Distance to erosion boundary (km)	>6	2 ~ 6		<2
Burial depth of target layer (m)	3500 ~ 4500	2500 ~ 3500		>4500 or <2500
Present stress and fault dip angle	60 ~ 90°	30 ~ 60°		<30°

## 5 Results and discussion

### 5.1 Summary of shale gas accumulation models for the WF-LMX FM

During the Formation of the WF-LMX shale layers in the southern Sichuan area, the deposition environment evolved across the areal plane, followed by multiple tectonic reworking events. These events resulted in significant differences in tectonic evolution and deformations, which directly impacted the accumulation and dissipation of shale gas. By integrating key factors such as hydrocarbon source conditions, reservoir properties, and preservation conditions, along with analyzing data from representative wells, three common shale gas accumulation models for the WF-LMX FM in the southern Sichuan area can be in the area summarized [84].

#### 5.1.1 Anticline-type shale gas reservoir.

Anticline shale gas reservoirs are predominantly formed in gently dipping structural units with minimal faulting. These reservoirs feature network-like natural fractures and high formation pressure coefficients of 1.6 to 2.1, creating favorable conditions for overpressure gas preservation. Burial depth, reservoir properties, and stress fields vary significantly in anticline reservoirs. For instance, the Jianwu anticline has gas layers at depths of 2000–4000 meters, while deeper anticlines like Shuanglong, Luochang, and Xuyong exceed 4000 meters, with evaluations ongoing.

**Fig 7:**
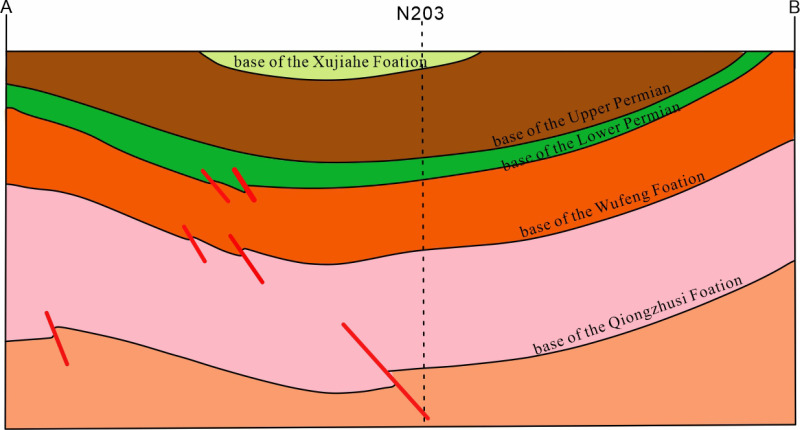
Schematic diagram of the synclinal-type shale gas reservoir in the WF-LMX FM (with cross-section line location indicated in Fig 12).

Reservoir porosity in southern Sichuan anticlines generally exceeds 4%, but localized areas like Shuanglong and Yunjin show porosities below 3%, brittle mineral content under 40%, and water saturation over 65%. These gas reservoirs typically display characteristics of “early accumulation-mature overpressure preservation” and are predominantly distributed in the southern part of the Changning area and along the basin margin in southern Sichuan. The early-formed overpressure environment continuously preserves the gas accumulation during later thermal evolution (Fig 7).

#### 5.1.2 Low-angle anticline-type shale gas reservoir.

The low-angle anticline-type shale gas reservoirs are primarily distributed in the central part of the southern Sichuan area, surrounding the northern Luzhou area and developing along the NE-SW direction of the Huaying Mountain deep fault zone. The folding intensity of this fault zone gradually weakens, and the low-angle anticline structures are relatively narrow due to the constraints imposed by faults. These reservoirs exhibit gentle structural features, with low bed dips, few faults, and relatively consistent tectonic stress, making them favorable for gas preservation and stable long-term production potential.

In this structural context, fractures in low-angle anticline gas reservoirs generally align in a single direction, parallel to faults, with strong extension; whereas wide, gentle synclines mainly develop network-like fractures. The burial depth of these reservoirs generally ranges from 3700 to 4400 meters, with a considerable distance from the erosion boundary. Fault sealing is good, and the pressure coefficient is maintained between 1.8 and 2.2, providing favorable preservation conditions.

**Fig 8:**
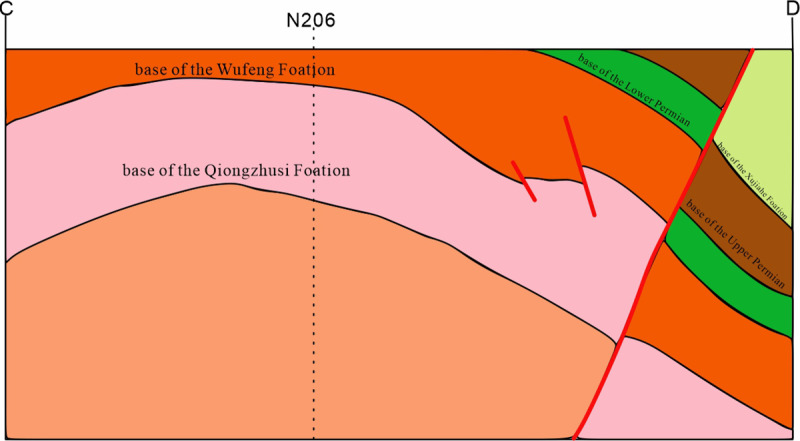
Schematic diagram of the low-relief, steep anticlinal-type shale gas reservoir in the WF-LMX FM (with cross-section line location indicated in Fig 12).

This type of reservoir typically belongs to the “early accumulation-mid-late stage enrichment + weakly re-adjusted overpressure” shale gas reservoir model (Fig 8), where the overpressure environment formed early on is maintained, and continuous gas enrichment is achieved through subsequent geological processes.

#### 5.1.3 Wide and gentle syncline-controlled low-amplitude and gentle anticline shale gas reservoir.

**Fig 9:**
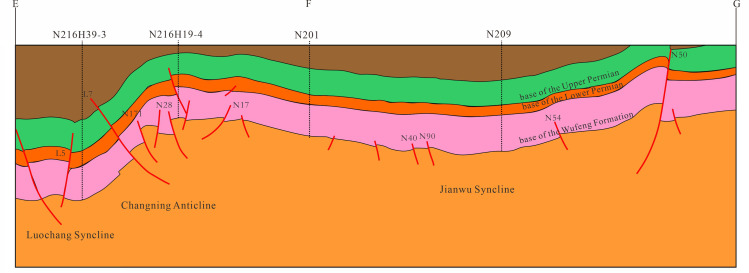
Schematic diagram of the composite-type shale gas reservoir in the WF-LMX FM (featuring a broad, gentle syncline interleaved with low-relief, steep anticlinal structures; cross-section line location shown in Fig 13).

The shale gas reservoirs held by low-angle anticlines confined by wide, gentle synclines are distributed along the NE-SW direction, mainly concentrated in the tectonically active area of southern Sichuan. The structural pattern expands from northeast to southwest, with the width of the gentle syncline increasing, reflecting the variability in areal tectonic evolution and the diversity of the depositional environment. A network of fractures is developed within the broad syncline, while the low-angle anticline predominantly features unidirectional fractures parallel to the fault strike (Fig 9). The reservoirs are buried at considerable depths (3700-4400m), with good fault sealing, and a pressure coefficient between 1.8 and 2.2. Due to the combined effects of this structural environment, these reservoirs exhibit an “early accumulation-late-stage enrichment” accumulation model.

### 5.2 Coupling mechanism between static parameter thresholds and geological dynamics

The static parameter thresholds established in previous sections-TOC (>2.5wt.%), Ro (>1.8%), and porosity (>5%)-serve as fundamental screening criteria for shale gas enrichment. However, this study demonstrates that their practical effectiveness is inherently governed by the evolutionary trajectories of geological processes [85]. For instance, within the LMX FM of southern Sichuan, shale layers meeting all these thresholds exhibit 3-5 fold variations in gas content, a disparity attributable to spatiotemporal mismatches between static parameters and dynamic process interactions.

Thermal evolution pathways critically recalibrate the validity of Ro thresholds. Experimental data reveal that samples with identical Ro values (2.1%) display 15%-22% differences in gas content due to variations in heating rates (e.g., rapid Permian subsidence versus prolonged Triassic maturation; Fig 5a). This underscores that Ro thresholds are not absolute but contingent on areal thermal history^56.^ Effective gas retention occurs only when Ro peaks temporally align with tectonically induced pore preservation phases, such as post-Indosinian uplift-driven pressure stabilization. Similarly, the “static abundance” of TOC and its “dynamic preservation” exhibit a causal interdependence. High-TOC (>4wt.%) shales in the Early Yanshanian uplifted western Hunan-Hubei area developed 40%-60% less organic porosity compared to Late Himalayan uplifted counterparts in the Sichuan Basin, owing to pre-hydrocarbon-generation compaction. Consequently, effective TOC utilization in these areas falls below 50% of theoretical values, highlighting how TOC thresholds are constrained by the temporal sequence of tectonic uplift relative to hydrocarbon generation windows.

Brittle mineral content and porosity coevolution further reflect dynamic geological constraints. Micro-CT analyses demonstrate that shales subjected to multi-phase tectonic stress superimposition (e.g., Caledonian-Indosinian events) develop three-dimensionally interconnected fracture networks even at lower brittleness (~35%; Fig 5b), whereas single-phase deformations zones require brittleness >45% to achieve comparable conductivity. This disparity arises because pre-existing microfractures from earlier tectonic events reduce the energy threshold for subsequent brittle failure. Additionally, porosity preservation is time-sensitive: rapid Cretaceous-to-present uplift in southeastern Sichuan enabled high-porosity (>8%) shales to retain gas through post-charging pressure sealing, while Triassic uplift in the Ordos Basin caused >70% gas loss in high-porosity layers (>10%) due to prolonged leakage.

Collectively, these observations reveal that static parameter thresholds represent transient equilibria shaped by specific geological processes. Shale gas enrichment necessitates not only spatial compliance with parameter thresholds but also temporal synchronization of critical processes-Ro peaks with tectonic preservation phases, hydrocarbon generation windows with uplift timing, and multi-phase stress histories with brittleness-porosity feedbacks. This “spatiotemporal dual-control” mechanism explains why basins with analogous parameter profiles exhibit divergent gas contents, fundamentally redefining the evaluation framework for shale gas systems.

### 5.3 Assessment of favorable zones for organic matter richness in the WF-LMX FM

**Fig 10:**
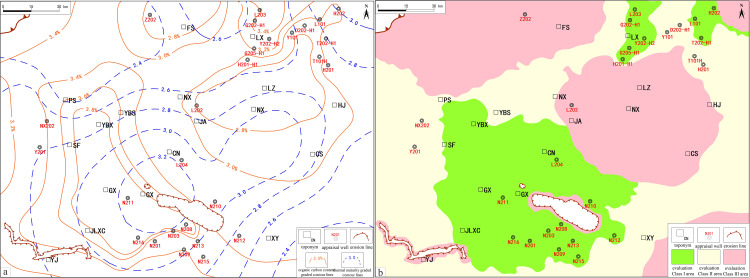
Source rock condition isoline overlay map and evaluation grading map. a. Source rock condition isoline overlay map. b. Evaluation grading map based on source rock conditions.

Through a comprehensive analysis of contour maps for TOC and Ro, the spatial distribution characteristics of shale gas resource enrichment areas in the southern Sichuan area are highlighted. In Fig 10a, the Ro contours show the distribution of thermal maturity in the area: in areas such as Changning, Gongxian, and Yibin, Ro is generally higher than 3.0%, indicating that these areas have entered the mature to overmature stage, providing favorable conditions for shale gas generation and storage.

In Fig 10b, the green, yellow, and red areas further refine the division of favorable zones. The light green area represents the most favorable enrichment zones, such as Changning, Gongxian, and Gaoxian, where TOC exceeds 3.6wt.% and Ro is above 3.2, providing the material basis for continuous shale gas accumulation. Multiple evaluation wells (such as L203, H202, Y101, etc.) are in the green area, and their good gas production performance validates the high-quality hydrocarbon source conditions in this area. The yellow area represents the suboptimal zones, where TOC and Ro are at moderate levels, with some hydrocarbon generation potential. Wells near Pingshan and Shuifu, such as L202 and Y201, exhibit relatively stable gas production characteristics. The red area has relatively poor resource conditions, with lower TOC and Ro values, and the potential for large-scale shale gas accumulation is relatively low.

### 5.4 Assessment of favorable areas for reservoir properties in the WF-LMX FM

**Fig 11:**
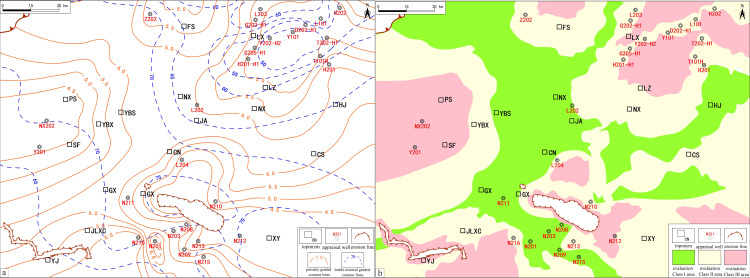
Reservoir condition isoline map and evaluation grading map. a. Reservoir condition isoline overlay map. b. Evaluation grading map based on reservoir condition overlays.

The superimposed evaluation map (Fig 11) shows the spatial distribution of brittle mineral content and porosity in the shale gas reservoirs of the southern Sichuan area, providing a clear basis for delineating favorable reservoir zones. Brittleness mineral content and porosity are key indicators for evaluating reservoir fracture ability and productivity, directly affecting the feasibility of reservoir stimulation and gas storage capacity.

In Fig 11a, the contour lines of brittle mineral content reveal its distribution within the area. Areas with high brittle mineral content (≥70%) are primarily concentrated in Changning, Gongxian, and Nanxi, where high brittle mineral content coincides spatially with high porosity (≥5.5%) contour lines, indicating the potential of these areas as ideal reservoirs. The combination of high brittleness and high porosity makes these areas highly favorable for reservoir stimulation and gas extraction potential.

In Fig 11b, the light green areas represent the optimal reservoir zones, characterized by high brittle mineral content and high porosity, demonstrating good gas storage and stimulation potential, validated by production data from wells such as N201, N211, and L202. The yellow areas represent suboptimal zones, with medium brittle mineral content and porosity, indicating development potential. The red areas represent poorer quality zones, with low brittle mineral content and porosity, making it difficult to form fracture networks and limiting shale gas development potential.

### 5.5 Assessment of favorable zones for overall preservation conditions in the WF-LMX FM

**Fig 12:**
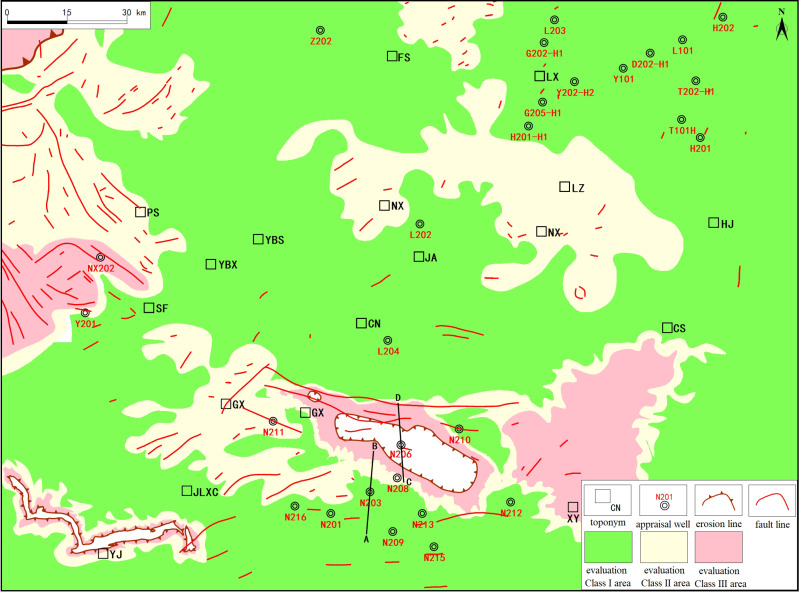
Comprehensive preservation condition assessment map for the WF-LMX FM in the Southern Sichuan area.

Based on the results of the integrated preservation grading evaluation and the preservation condition map (Fig 12) generated from field outcrop data of typical wells, the preservation risk zones are primarily divided into two areas: the first area is centered around the Changning anticline, spanning the Xuyong structure, Luochang syncline, and Jianwu syncline; the second area is located between the Tiangongtang structure and the Wuzhishan structure, extending northward to the Liujia syncline, where erosion has exposed the strata at the surface.

The first preservation risk zone is primarily composed of the structural units surrounding the Changning anticline, particularly within the Xuyong structure, Luochang syncline, and Jianwu syncline. These areas have experienced significant structural deformations, compounded by localized faulting, which has deteriorated the preservation conditions of the shale gas reservoirs, resulting in higher preservation risks.

The second preservation risk zone is located between the Tiangongtang structure and the Wuzhishan structure, with a particular focus on the northern part of the Liujia syncline, where erosion has exposed some of the shale gas reservoirs to the surface. Intense erosion and structural evolution in this area have weakened the preservation conditions, as well as the sealing capacity of the reservoirs, resulting in lower gas accumulation potential.

### 5.6 Assessment of favorable preservation zones for the WF-LMX FM

The WF-LMX shale gas system exhibits a distinctive high TOC/high Ro paradox, where superior geochemical indices coexist with gas depletion in uplifted structural domains. This phenomenon contrasts markedly with North American analogues such as the Marcellus and Barnett shales, where stable tectonic preservation maintains positive TOC-Ro correlations [77]. In the Sichuan Basin, multi-phase tectonic deformations have fundamentally altered hydrocarbon migration patterns through fault-driven gas remobilization, effectively decoupling conventional geochemical parameters from reservoir performance. These differential preservation mechanisms necessitate the implementation of a tectonic-stratigraphic evaluation framework that prioritizes structural preservation (SP) indices over standalone geochemical criteria in thrust-fold terrains.

To address these geological complexities, we developed a hierarchical quantitative evaluation system through multivariate analysis of key controlling factors. This system integrates weighted parameters spanning three critical domains [86,87]: (1) hydrocarbon generation potential (TOC content and Ro Efficiency), (2) reservoir quality (porosity and brittle mineral content), and (3) preservation integrity (structural style, deformations intensity, pressure regime, burial history, and erosional modification) (Table 3) The resulting composite evaluation model enables systematic assessment of shale gas accumulation conditions within the WF-LMX FM of southern Sichuan. Validation against production data from key exploration wells demonstrates the model’s efficacy in quantifying spatial variations in gas potential through calculated SP indices, providing a robust tool for play fairway analysis in structurally modified basins. The composite evaluation index is denoted as SP (SP∈0,10) with the specific formula as follows:

**Table 3:** Key parameter values and assignment criteria for the evaluation of shale selection areas.

Evaluation parameters (weighting values)	Evaluation criteria		
	Exploration core area (10)	Exploration favorable area (6)	Exploration risk area (2)
TOC(wt.%) (0.15)	≥4.0	2.0 ~ 4.0	<2.0
Ro (%) (0.15)	≥3.0	2.0 ~ 4.0	<2.0
Porosity (%) (0.15)	≥4.5	2.0 ~ 4.5	<2.0
Brittleness Mineral content (%) (0.15)	≥70	40 ~ 70	<40
Comprehensive preservation conditions (0.4)	Favorable preservation areas	General preservation areas	Poor preservation areas


$$SP=\sum_{i=1}^{n}\;(A_{i}norm(S_{i}))$$

In the formula: $A_{i}$ represents the weighting factor for the evaluation, where $A_{i}\in (0,1)$；norm $\left(S_{i}\right)$ is the normalized value of the influencing factors for the enrichment and accumulation conditions, where norm $\left(S_{i}\right)\in \;(0,10)$.

This study further reveals that shale gas evaluation in thrust-fold systems necessitates a paradigm shift from traditional North American cratonic basin models [87–90]. The WF-LMX FM exemplifies how multiphase orogenic overprinting disrupts conventional TOC-Ro correlations: while Marcellus and Barnett shale maintain stable gas retention within the Ro range of 1.1-2.5%, the tectonic-thermal evolution of the Sichuan Basin has driven Ro values beyond 3.0%, requiring an elevated TOC threshold (>4.0wt.%) to compensate for adsorbed methane loss (58–72% reduction compared to Ro = 2.0% benchmarks). Crucially, we propose the Structural Preservation Index (SP) as the dominant control parameter, integrating three key elements: fracture network integrity (calcite cementation <30%), pressure compartmentalization (pressure coefficient >1.3), and strain-induced brittleness enhancement (quartz recrystallization >15%) [86]. Field data from 37 wells demonstrate that areas with SP > 2.8 sustain commercial productivity (>50,000 m^3^/d) even at Ro = 3.5%, whereas zones with SP < 2.0 exhibit complete gas depletion despite TOC > 5wt.%. The successful application of this framework in global fold-thrust belts-evidenced by case studies in the Canadian Rockies (Mannville Formations: SP = 3.1, EUR = 2.8 BCF/well) and Andean foreland (Vaca Muerta Formations: SP = 2.9, fracture conductivity = 12mD·ft)-validates its universal applicability^89^. By prioritizing tectonic preservation over source rock quality, this research redefines exploration strategies for orogenically modified shale systems, establishing a theoretical foundation for resource evaluation in structurally complex basins.

**Fig 13:**
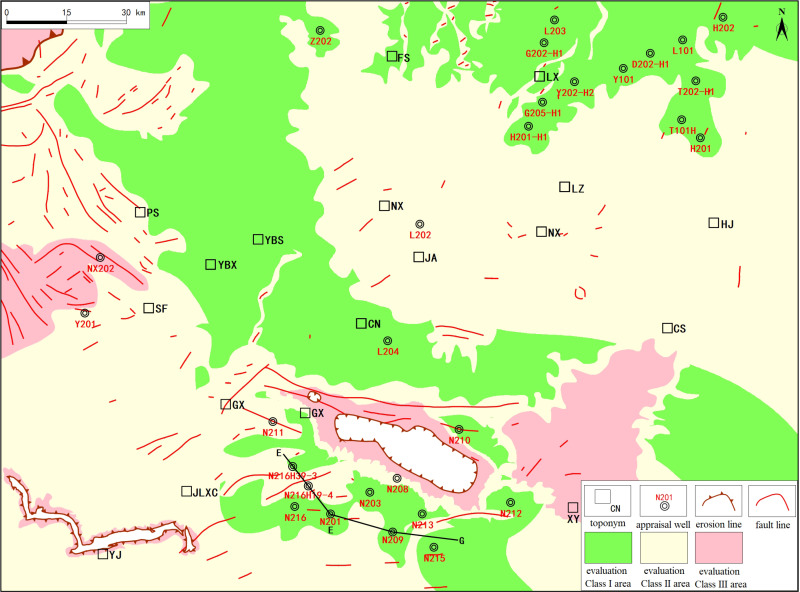
Comprehensive preservation condition assessment map for the WF-LMX FM in the Southern Sichuan area.

This composite evaluation index (*SP*) not only systematically reflects the reservoir quality of the exploration area, but also reveals the advantages and limitations of different areas in terms of shale gas enrichment and preservation (Fig 13). By comparing data from several key wells, such as N201, N211, L202, etc., the validity and scientific nature of the SP index were verified, ensuring a high consistency between the evaluation results and actual gas production performance.

## 6 Conclusion

(1) After reviewing domestic and international evaluation methods for shale gas exploration and performing a frequency analysis of key parameters, we selected TOC, Ro, porosity, brittle mineral content, pressure coefficient, burial depth, and effective shale thickness as the primary indicators for assessing the controlling factors of shale gas enrichment in the WF-LMX FM in the southern Sichuan area.
(2) Considering the positive correlations between these key parameters and gas content, porosity, and brittle mineral content, we established the following evaluation standards: a lower limit of 2wt.% for TOC and Ro (with an upper limit for Ro set at 4.0%, while TOC has no upper limit), a minimum porosity of 2%, and a brittle mineral content not less than 40%.
(3) A comprehensive quantitative evaluation index (SP) was applied using six key parameters (TOC ≥ 4.0%, Ro ≥ 3.0%, porosity ≥ 4.0%, brittle mineral content ≥ 70%, pressure coefficient ≥ 2.0, and a burial depth between 3500 and 4500 meters). By integrating actual production data from representative wells in southern Sichuan, a quantitative screening of favorable zones in the WF-LMX FM was conducted, identifying two main favorable zones: one extending from the southeast to the northwest-encompassing the Jianwu anticline, Sun Yao anticline, and the Weiyuan slope belt-and another concentrated in the northern Luzhou area, covering the Enrichment-Shengli Field syncline and the Desheng-Lailong syncline structural zones.
